# Gene expressions between obligate bamboo-eating pandas and non-herbivorous mammals reveal converged specialized bamboo diet adaptation

**DOI:** 10.1186/s12864-023-09111-z

**Published:** 2023-01-16

**Authors:** Jinnan Ma, Liang Zhang, Fujun Shen, Yang Geng, Yan Huang, Honglin Wu, Zhenxin Fan, Rong Hou, Zhaobin Song, Bisong Yue, Xiuyue Zhang

**Affiliations:** 1 Key Laboratory of Bio-Resources and Eco-Environment, Ministry of Education, College of Life Sciences, Sichuan University, No.24 South Section 1, Yihuan Road, Chengdu, 610065 China; 2 College of Continuing Education, Yunnan Normal University, Kunming, 650092 China; 3 The Sichuan Key Laboratory for Conservation Biology of Endangered Wildlife, Chengdu Research Base of Giant Panda Breeding, Chengdu, 610081 China; 4 Sichuan Key Laboratory of Conservation Biology On Endangered Wildlife, College of Life Sciences, Sichuan University, No.24 South Section 1, Yihuan Road, Chengdu, 610065 China; 5 China Conservation and Research Center for the Giant Panda, Wolong, 623006 Sichuan China

**Keywords:** Giant panda, Red panda, Gene expression pattern, Dietary shift, Convergence

## Abstract

**Background:**

It is inevitable to change the function or expression of genes during the environmental adaption of species. Both the giant panda (*Ailuropoda melanoleuca*) and red panda (*Ailurus fulgens*) belong to Carnivora and have developed similar adaptations to the same dietary switch to bamboos at the morphological and genomic levels. However, the genetic adaptation at the gene expression level is unclear. Therefore, we aimed to examine the gene expression patterns of giant and red panda convergent specialized bamboo-diets. We examined differences in liver and pancreas transcriptomes between the two panda species and other non-herbivorous species.

**Results:**

The clustering and PCA plots suggested that the specialized bamboo diet may drive similar expression shifts in these two species of pandas. Therefore, we focused on shared liver and pancreas DEGs (differentially expressed genes) in the giant and red panda relative to other non-herbivorous species. Genetic convergence occurred at multiple levels spanning carbohydrate metabolism, lipid metabolism, and lysine degradation. The shared adaptive convergence DEGs in both organs probably be an evolutionary response to the high carbohydrate, low lipid and lysine bamboo diet. Convergent expression of those nutrient metabolism-related genes in both pandas was an intricate process and subjected to multi-level regulation, including DNA methylation and transcription factor. A large number of lysine degradation and lipid metabolism related genes were hypermethylated in promoter regions in the red panda. Most genes related to carbohydrate metabolism had reduced DNA methylation with increased mRNA expression in giant pandas. Unlike the red panda, the core gene of the lysine degradation pathway (AASS) doesn’t exhibit hypermethylation modification in the giant panda, and dual-luciferase reporter assay showed that transcription factor, NR3C1, functions as a transcriptional activator in AASS transcription through the binding to AASS promoter region.

**Conclusions:**

Our results revealed the adaptive expressions and regulations of the metabolism-related genes responding to the unique nutrients in bamboo food and provided data accumulation and research hints for the future revelation of complex mechanism of two pandas underlying convergent adaptation to a specialized bamboo diet.

**Supplementary Information:**

The online version contains supplementary material available at 10.1186/s12864-023-09111-z.

## Background

Understanding how species have adapted to their environment has long interested evolutionary biologists [1]. Adaptive phenotypes can result from changes in protein-coding sequences that affect protein structure and function [2–4], and from gene expression alterations [5]. Gene expression is an intermediate phenotype that links DNA sequence and physiological traits [6]. Alterations in gene expression are more likely to cause adaptive changes in morphology and development than changes in protein sequences [7]. For example, environmental adaptations in humans are tenfold more likely to affect gene expression than amino acid sequences [8]. Adaptive phenotypes driven by alterations in gene expression patterns are more flexible than changes in amino acid sequence, and species can temporarily adapt to the environment by regulating gene expression [9]. Therefore, it is necessary to examine gene expression changes that occur during environmental adaptation.

Diet plays a pivotal role in the evolutionary history of animals [10]. Specialization of diets have resulted in the evolution of similar morphological, physiological, behavioral and biochemical adaptations [11–15]. However, molecular studies focused on the convergence of diets and the adaptations of specific taxa are rare. The giant panda and red panda belong to different families and both are specialized herbivores that independently evolved from meat-eating ancestors, making them ideal models for studying convergent evolution [16]. Of particularly interest is how the pandas obtain enough nutrition from low-nutrition and high-fiber bamboo with a typical Carnivore digestive tract. Previous research has indicated that both pandas have developed some similar features to adapt to the same dietary switch to bamboo, such as false fingers and pseudogenization of umami receptor gene [16]. In addition, of 70 adaptively convergent genes from the two panda genomes, only seven genes were related to nutrient utilization for a strictly bamboo diet [16]. However, this is far from achieving a comprehensive understanding of the genetic basis of adaptive evolution for the two panda species. Similarly, some phenotypic differences due to gene expression changes are unable to be directly explained by genome analyses [17] and changes of transcriptions may play an independent role in adaptive evolution [6]. We thus aimed to examine the nutrition-related gene expression patterns and their regulations of giant and red panda convergent specialized bamboo-diets. DNA methylation, as a major epigenetic factor, plays a critical role in regulating the expression of genes [18], and thus has important role in environmental adaptation and phenotypic shaping of species [19–21]. DNA methylation patterns are not static but can be altered by diet and multiple factors [22–25]. Therefore, we compared liver and pancreas tissue transcriptomic data from bamboo-feeding pandas and non-bamboo-feeding mammals (Table 1), and trying to offer an example of transcription-scale analyses for detecting convergent evolution. Combined with methylation data, the regulation mechanism of the convergent expression of nutrition metabolism-related genes in the liver and pancreas was further analyzed.

**Table 1:** Summary of mammals and RNA-seq libraries used for RNA-seq in this study

Scientific name	Common name	Order	Family	Genus	Number of RNA-seq libraries		Source^*^
					Liver	Pancreas	
*Ailuropoda melanoleuca*	Giant panda	Carnivora	Ursidae	*Ailuropoda*	4	4	This study
*Ailurus fulgens*	Red panda	Carnivora	Ailuridae	*Ailurus*	4	4	This study
*Mustela putorius furo*	Ferret	Carnivora	Mustelidae	*Mustela*	3	3	This study
*Canis lupus familiaris*	Domestic dog	Carnivora	Canidae	*Canis*	3	4	SRA
*Felis catus*	Cat	Carnivora	Felidae	Felis	2	-	SRA
*Homo sapiens*	Human	Primates	Hominidae	*Homo*	3	2	SRA
*Rattus norvegicus*	Rat	Rodentia	Muridae	*Muridae*	3	2	SRA
*Mus musculus*	Mouse	Rodentia	Muridae	*Mus*	4	2	SRA

^*^ RNA-seq libraries for liver and pancreas of 6 species were downloaded from NCBI Short Read Archive (SRA)

## Results

### RNA sequencing and sample cluster analysis

RNA extracted from liver and pancreas tissue from adult giant pandas, adult red pandas, and ferrets was sequenced using Illumina HiSeq2000. Sequencing generated 57.5G clean data for giant panda samples, 61.4G clean data for red panda samples, and 38.2G clean data for ferret samples. The sequences of each sample were the aligned to the genome sequence of the respective species. RNA-seq read alignment efficiency varied from 85 to 99% (Table S1).

**Fig. 1:**
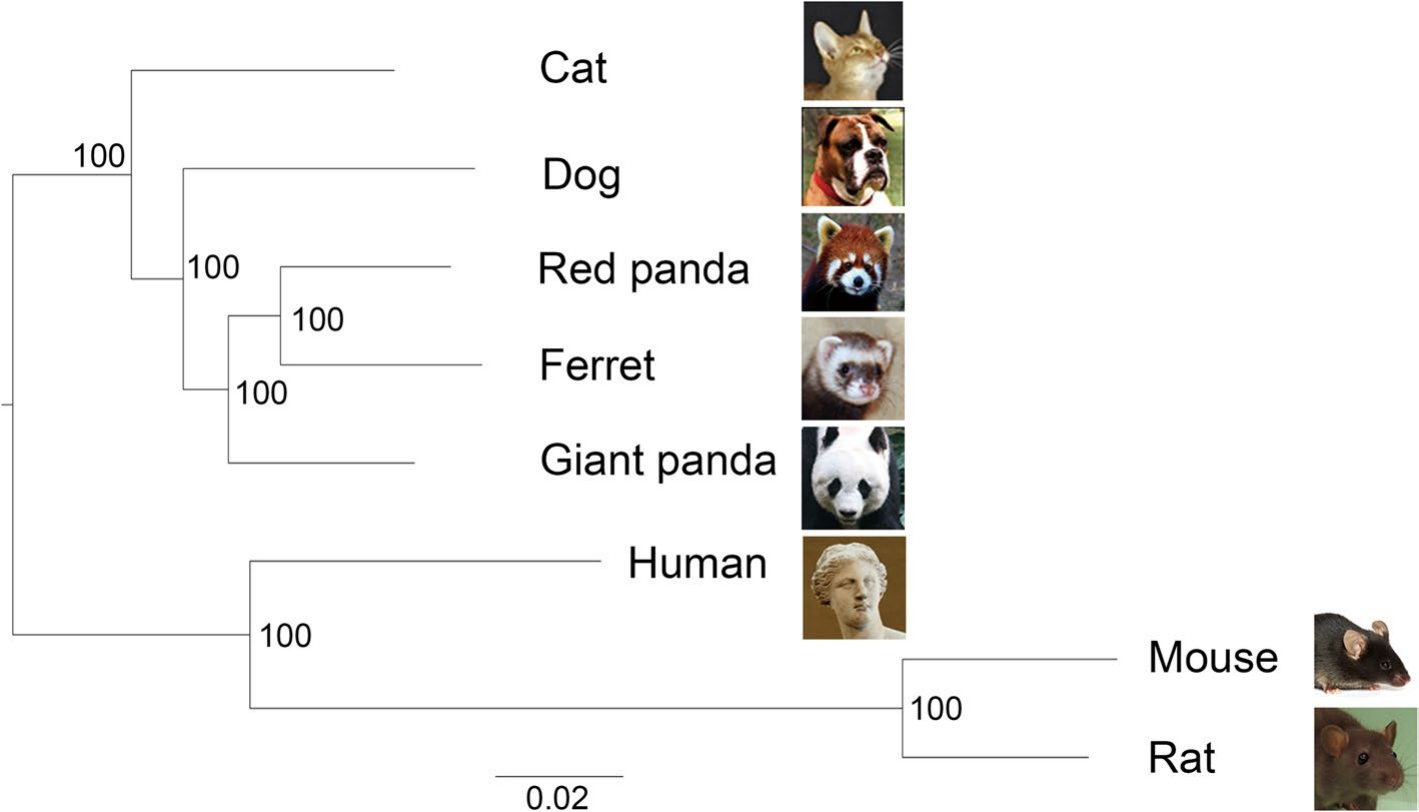
Maximum likelihood phylogeny of the eight species in this study based on 1:1 single-copy orthologues coding sequences. The numbers in the nodes represent the bootstrap values. The scale bar indicates the number of substitutions per site

We identified 9,219 and 9,546 1:1 single-copy orthologue genes from liver samples of eight species and pancreas samples of seven species, respectively. The corresponding sets of single-copy orthologues were used for comparisons among species within the liver and pancreas. The amino acid sequences of 9,219 single-copy genes were used to construct the ML phylogenetic tree of all species covered in this study. The red panda and ferret were clustered together with a high support value, and they were the sister group to the giant panda (Fig. 1). The topology corresponded with a previous phylogenic study [16], showing giant panda and red panda belong to different phylogenetic clades.

**Fig. 2:**
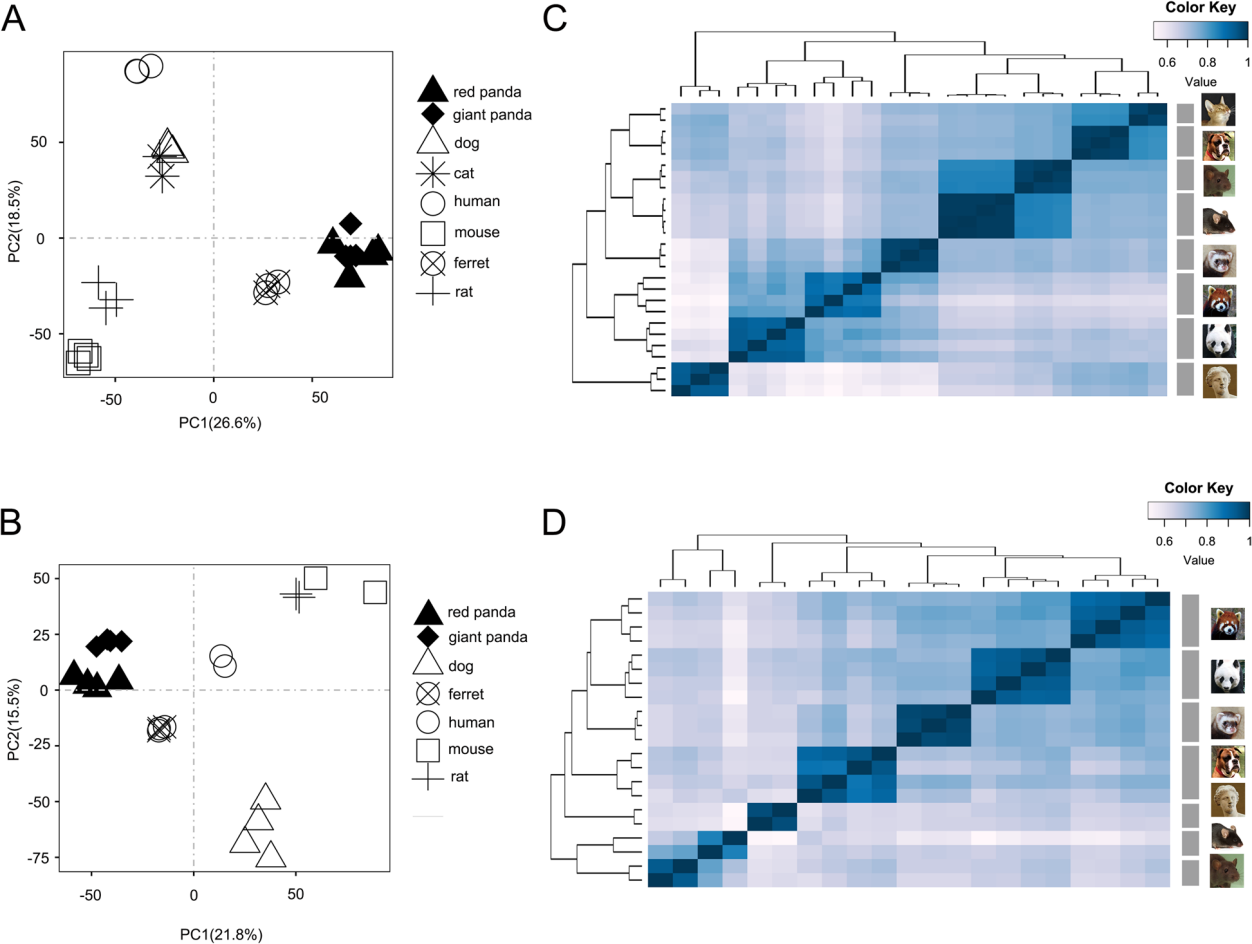
PCA and clustering analyses of the expression for each tissue based on different gene sets. **A** PCA of the log-transformed normalized expression levels of all orthologs across liver samples. Species are represented by point shape. **B** PCA of the log-transformed normalized expression levels of all orthologs across pancreas samples. Species are represented by point shape. **C** Clustering of liver samples based on log-transformed normalized expression values. Distance between samples was measured by Spearman's rank correlation coefficient. **D** Clustering of pancreas samples based on log-transformed normalized expression values. Distance between samples was measured by Spearman's rank correlation coefficient

To obtain an initial overview of transcription patterns, we performed PCA and clustering analyses of the expression for each tissue based on different gene sets of 1:1 single-copy orthologues (Fig. 2). The PCA analyses clearly separated the data according to species, and the biological replicates were clustered together (Fig. 2A and B). The Spearman correlation relationship analyses also indicated a similar species-specific expression pattern (Fig. 2C and D). Notably, both panda species were grouped together and separated from other non-herbivorous animals in both PCA and clustering analyses, although red panda and ferret were evolutionary monophyletic [16]. Such a pattern of expression was therefore assumed to be correlated with adaptation to the obligate bamboo diet occupied by both panda species, without evolutionary relatedness.

### Transcriptomic profiles of liver

**Fig. 3:**
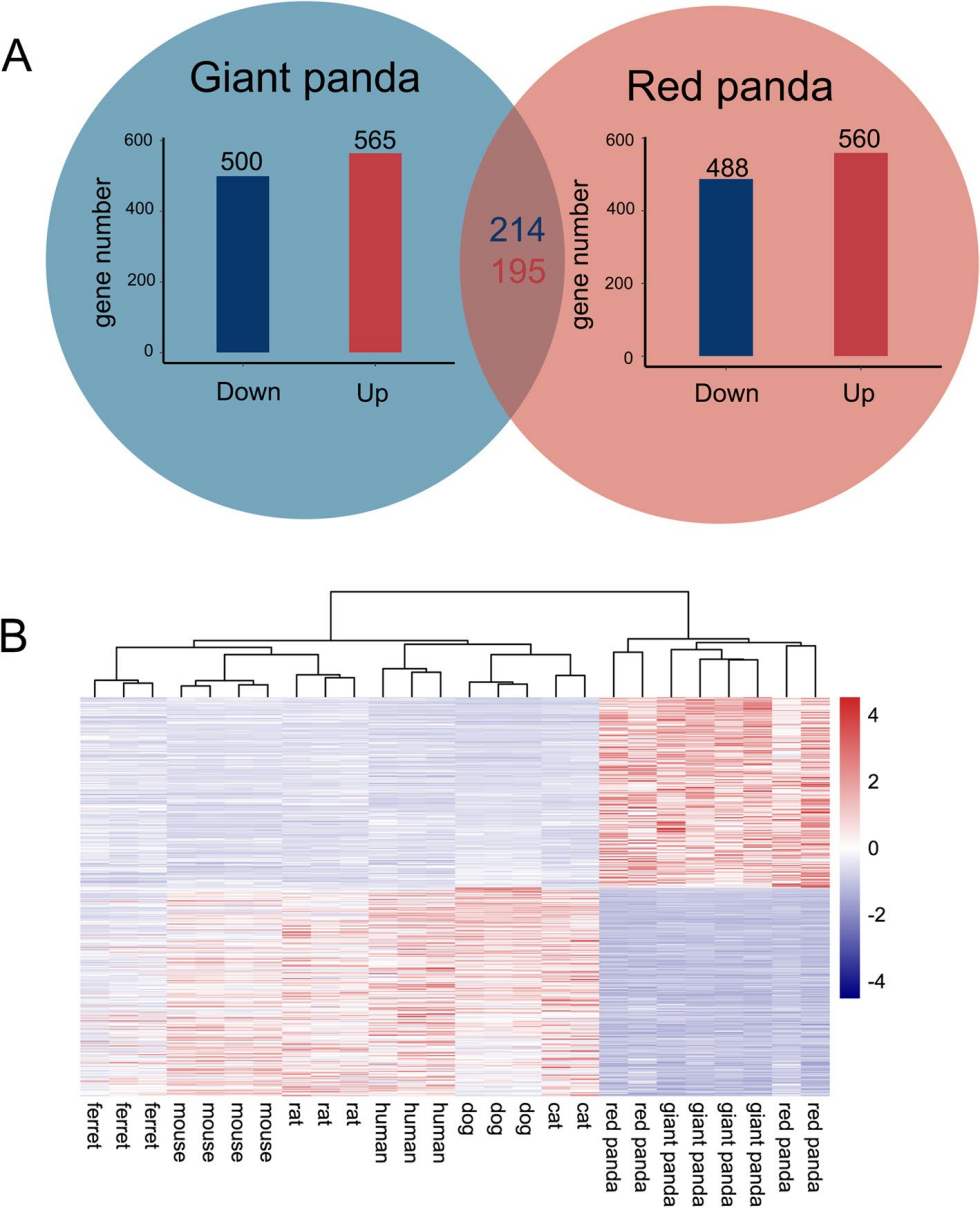
Transcriptional patterns in liver. **A** Bar plots in blue and red circles indicate numbers of shared DEGs in giant panda and red panda relative to other non-herbivorous species in liver, respectively. Numbers in red and blue indicate shared up- and down-regulated DEGs in both panda species. **B** Heat map plot of shared DEGs in both panda species using log-transformed normalized expression value of genes across liver samples by adopting hierarchical clustering method

We identified differences in liver DEGs of the pandas and the other six species using edgeR to detect diet-related gene expression shifts. Volcano plots of pairwise comparisons between species is illustrated in Figure S1A. A total of 565 shared genes were up-regulated and 500 shared genes were down-regulated in the giant panda compared to the other seven species. Shared genes between the red panda and the other seven species totaled 1,048 DEGs, with 560 up-regulated and 488 down-regulated DEGs. Among these DEGs, 214 down-regulated and 195 up-regulated DEGs were shared by the panda species, which were identified as convergent expression genes (Fig. 3A and Table S2). Hierarchical clustering was performed on these 409 DEGs, where giant and red panda samples were clustered into one group and the other seven species were clustered into another group (Fig. 3B). This highlights the expression level of DEGs in the bamboo diet group compared to the non-bamboo diet group.

**Fig. 4:**
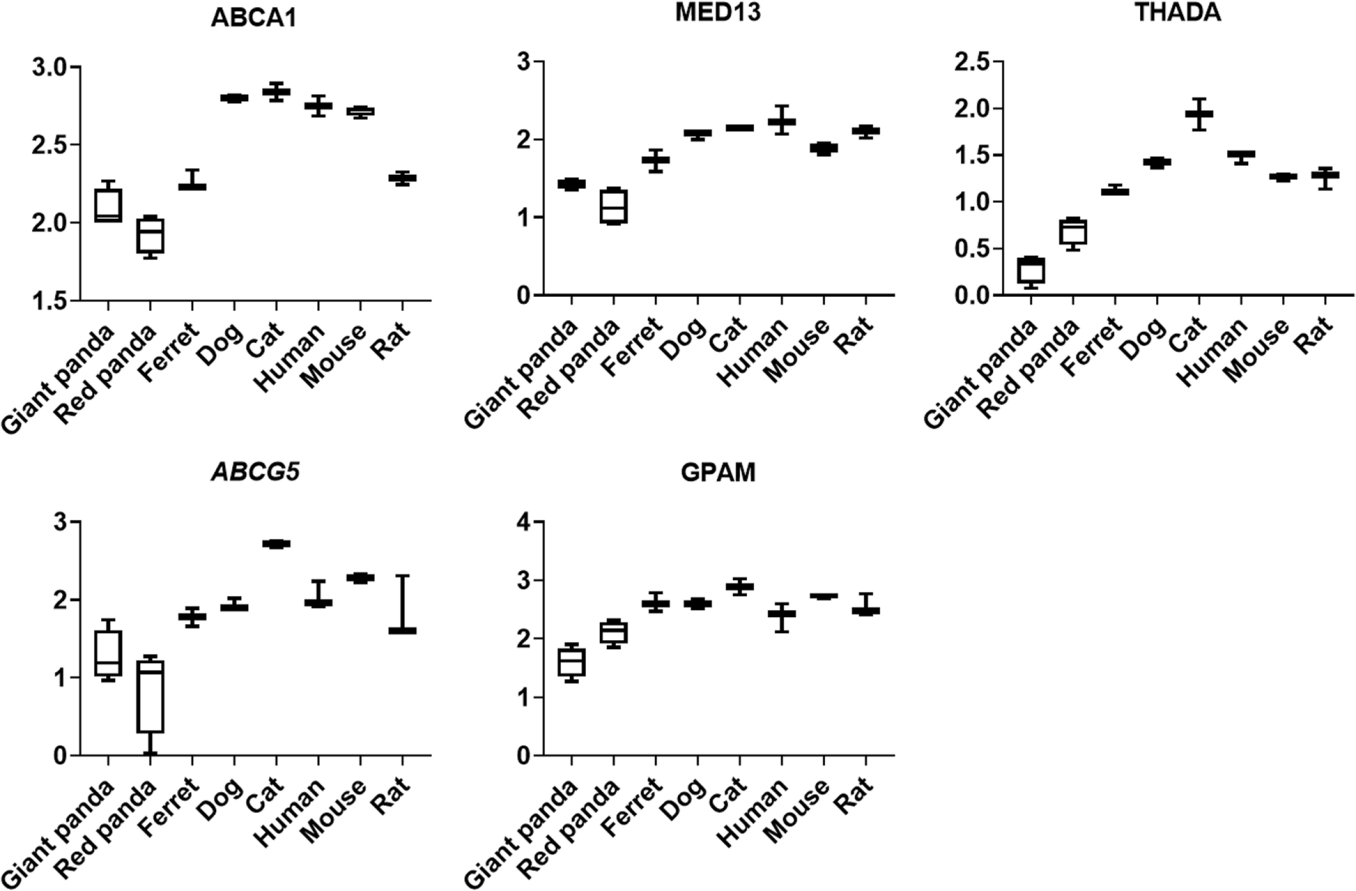
The expression tendency of DEGs associated with lipid metabolism in liver samples. Y-axis represents log-transformed normalized expression levels. Boxplot edges indicate the 25th and 75th percentiles, and whiskers indicate non-outlier extremes

**Fig. 5:**
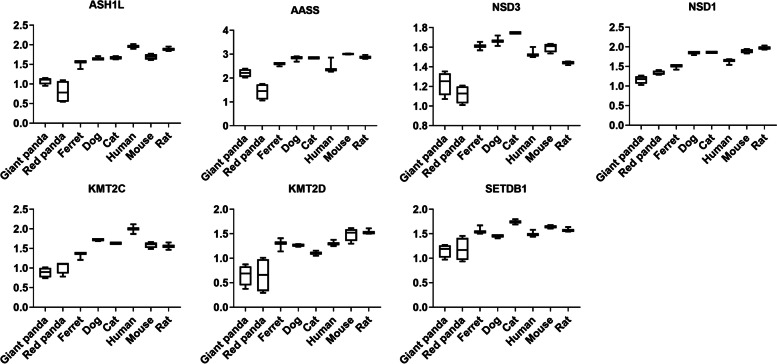
The expression tendency of DEGs associated with lysine degradation in liver samples. Y-axis represents log-transformed normalized expression levels. Boxplot edges indicate the 25th and 75th percentiles, and whiskers indicate non-outlier extremes

We performed GO category and KEGG pathway enrichment analyses to gain insights into the biological roles of the DEGs shared by the panda species. We found that 214 down-regulated genes were significantly enriched in 266 GO categories (Tables S4) and 8 KEGG pathways (Tables S5). These terms were involved in nutrient utilization, including lipid homeostasis (GO:0,055,088, *P* = 1.54E-02), cholesterol efflux (GO:0,033,344, *P* = 3.92E-02), and lysine degradation (map00310, *P* = 2.48E-06). The shared down-regulated DEGs associated with lipid metabolism were *ABCA1*, *MED13*, *THADA*, *ABCG5*, *GPAM* (Fig. 4 and Table S2) and shared down-regulated DEGs associated with lysine degradation in the liver were *ASH1L*, *AASS*, *NSD3*, *NSD1*, *KMT2C*, *KMT2D*, *SETDB1* (Fig. 5 and Table S2). Panda up-regulated genes were enriched in 285 GO terms, which mostly significantly associated with the immune system and developmental process (Table S6). These 195 up-regulated genes were significantly enriched in 23 KEGG terms involved in the similar biological processes (Table S7). Genes involved in carbohydrate metabolism and energy production (*PIK3CD*, *NDUFS4*, *TMEM126B*, *COQ4*) were also enriched in significantly up-regulated genes (Table S2).

### Transcriptomic profiles of pancreas

**Fig. 6:**
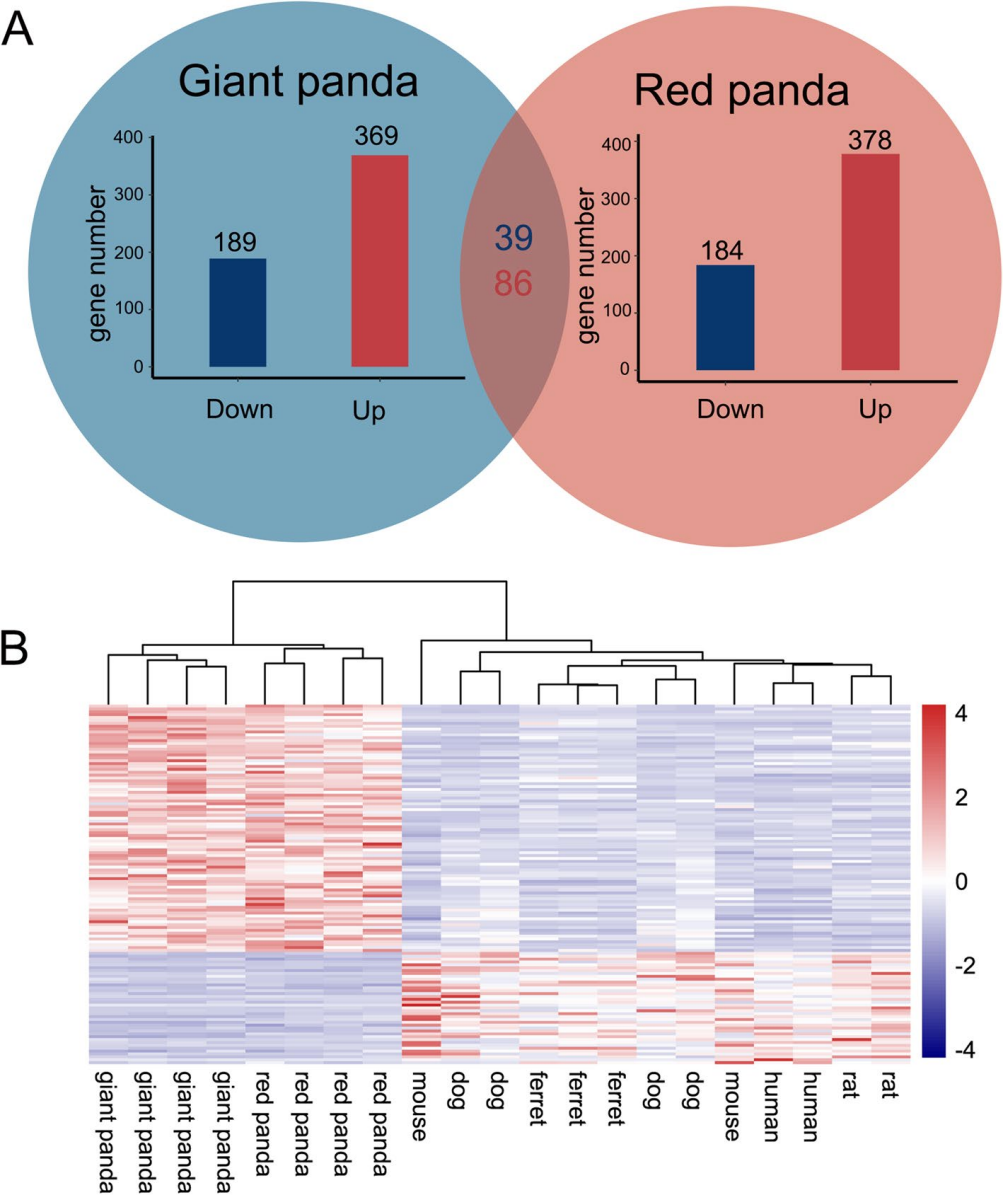
Transcriptional patterns in pancreas. **A** Bar plots in blue and red circles indicate numbers of shared DEGs in giant panda and red panda relative to other non-herbivorous species in pancreas, respectively. Numbers in red and blue indicate shared up- and down-regulated DEGs in both panda species. **B** Heat map plot of shared DEGs in both panda species using log-transformed normalized expression value of genes across pancreas samples by adopting hierarchical clustering method

With the same experimental design, we tested 21 pancreas samples from seven species. There were 558 DEGs (189 down-regulated and 369 up-regulated) shared by the giant panda and the five other species and 562 DEGs (184 down-regulated and 378 up-regulated) shared by the red panda and the five other species (Figure S1B). By combining these DEGs, we found 86 up-regulated and 39 down-regulated DEGs were convergent expression genes in both panda species (Fig. 6A and Table S3). The hierarchical clustering of pancreas DEGs showed a similar pattern to liver DEGs, with giant panda and red panda samples clustered away from other samples (Fig. 6B), suggesting that both panda species have similar expression profiles.

**Fig. 7:**
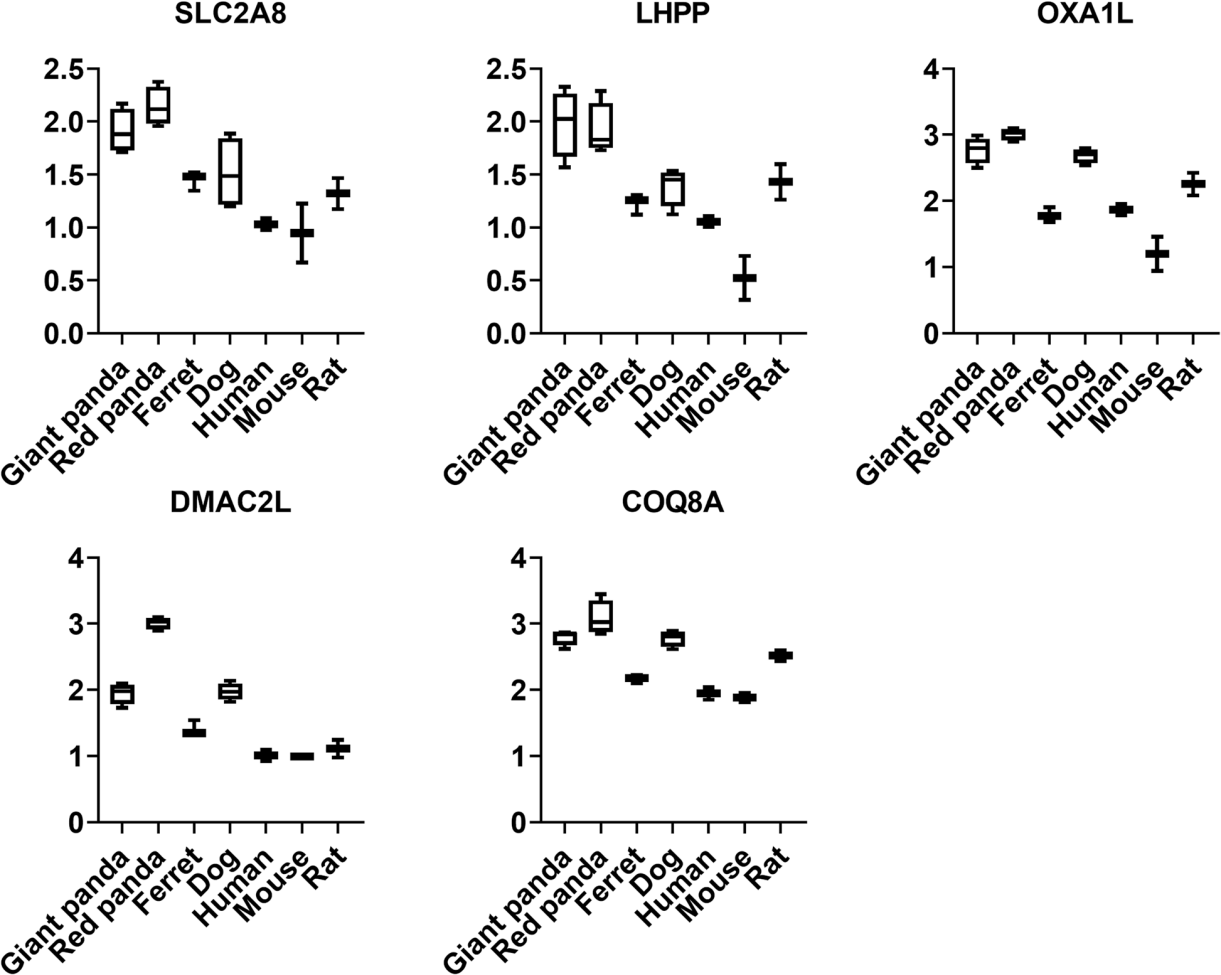
The expression tendency of DEGs associated with carbohydrate metabolism and respiratory electron transport in pancreas samples. Y-axis represents log-transformed normalized expression levels. Boxplot edges indicate the 25th and 75th percentiles, and whiskers indicate non-outlier extremes

GO analysis discovered 96 functional categories enriched in the 39 down-regulated panda-shared DEGs, including terms main related to nucleic acid metabolic process (e.g. 3'-5'-exoribonuclease activity (GO:0,000,175); nucleotide-excision repair, DNA damage recognition (GO:0,000,715); exoribonuclease activity (GO:0,004,532)) (Table S8). The 39 shared down-regulated genes were significantly enriched in five KEGG pathways (Table S9). Genes associated with lipid metabolism (*LOC100477302*) displayed signatures of down-regulation (Table S3). 86 up-regulated DEGs of the pancreas were significantly enriched in 98 GO categories (Tables S10) and 18 KEGG pathways (Tables S11), these terms were mainly related to basic biological processes (e.g. ribosomal large subunit biogenesis (GO:0,042,273), meiotic nuclear division (GO:0,140,013), Spliceosome (map03040)). Similar to liver, carbohydrate metabolism and respiratory electron transport related-genes (*SLC2A8*, *LHPP*, *OXA1L*, *DMAC2L*, *COQ8A*) were also found to be converging in high expression (Fig. 7 and Table S3).

To further confirm the reliability of results, the expressions of five carbohydrate metabolism and respiratory electron transport related-genes were verified by real-time quantitative PCR. As shown in Figure S2, the results of qRT-PCR revealed similar expression tendency. Despite some quantitative differences at the expression level, the result of qRT-PCR indicated that the convergent expression of nutrition metabolism-related genes were reliable.

### Promoter methylation patterns of convergently expressed nutrition metabolism-related genes in both pandas

In order to further analyze the regulation mechanism of the convergent expression of nutrition metabolism-related genes in the liver and pancreas, we explored from two aspects of transcription factors and DNA methylation respectively. Both transcription factors and DNA methylation can act on the promoter region to regulate gene expression. First of all, we tested the promoter methylation levels of the convergently expressed nutrition metabolism-related genes in both pandas, and compared them with different species (Figure S3). Studies demonstrated that the DNA methylation status of gene promoter and its mRNA expression was usually inversely correlated [26]. In the liver, the promoter methylation levels of a large number of shared down-regulated lysine degradation (*AASS*, *ASH1L*, *NSD3*, *NSD1*, *KMT2C*, *KMT2D*) and lipid metabolism related genes (*ABCA1*, *MED13*, *THADA*, *GPAM*) in the red panda were significantly higher than those of human and mice. Only two lysine degradation related genes (*KMT2C*, *KMT2D*) exhibited reduced methylation in promoters and increased gene expression in the giant panda. In the pancreas, all shared up-regulated genes (*SLC2A8*, *LHPP*, *OXA1L*, *DMAC2L*) involved in carbohydrate metabolism exhibited lower methylation in the giant panda comparing to human, and only *OXA1L* in the red panda represented the same methylation pattern.

### Regulation of AASS transcriptional activity by NR3C1

We want to know whether convergently expressed nutrition metabolism-related genes were interacting with specific transcription factors to regulate expression. Through published papers, we identified and integrated the transcription factors of shared adaptive convergence DEGs, of which only eight gene transcription factors have been reported (Table S12). *AASS* is the core gene in the pathway of the catabolism of L-lysine. We found that the expression of *AASS* and its transcription factor *NR3C1* (glucocorticoid receptor) in the giant pandas were lower than that of other 6 non-bamboo-feeding mammals. Previous study showed that *AASS* was regulated directly or indirectly by glucocorticoid receptor [27], and no methylomic change of *AASS* promoter was found among giant panda and other species comparisons (Figure S3A). We next asked whether the down-regulated *AASS* that we had identified in giant panda liver was regulated by NR3C1 in the cell line models.

**Fig. 8:**
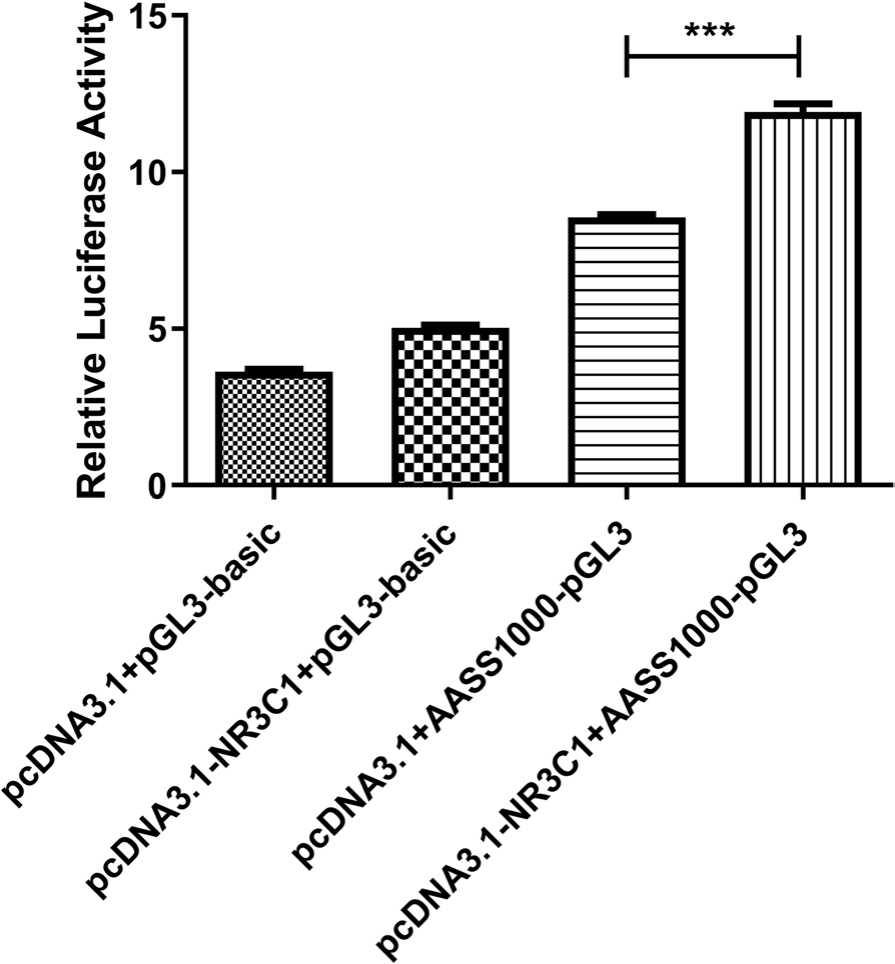
NR3C1 acts as an activator of AASS promoter activity. *** *P*<0.001

First, a total of eight NR3C1 binding motifs that were strongly associated with the *AASS* promoter were predicted on the JASPAR online website (http://jaspar.genereg.net/), indicating *AASS* were necessary for transcriptional regulation by NR3C1 (Table S13). Then, we performed luciferase assays. The NR3C1 expression vector was co-transfected along with the AASS promoter luciferase reporter plasmids into 293 T cells. Firefly luciferase activity was normalized based on Renilla luciferase activity. All reporter assays were repeated at least five times (Table S14). As shown in Fig. 8, co-transfection of AASS1000-pGL3-basic reporter plasmid and NR3C1 expression plasmid significantly increased the activity of AASS promoter. This result demonstrated that NR3C1 functions as a transcriptional activator in AASS transcription through the binding to AASS promoter region.

## Discussion

Despite belonging to the order Carnivora, the giant panda and the red panda are specialized herbivores making them an ideal model to study adaptive evolution in dietary shift and specialization [28]. Our study examined the gene expression changes that have occurred during the adaptation of pandas to a specialized bamboo diet. Indeed, both the dendrogram and PCA plots support the gene expression convergence of these two species of pandas.

### Genes associated with carbohydrate metabolism and energy production

Liver and pancreas are important sites for carbohydrate metabolism. Two carbohydrate metabolism related genes, *PIK3CD* and *SLC2A8*, showed adaptive convergence up-regulation in liver and pancreas, respectively (Table S15). PIK3CD regulates glucose uptake via PI3K-dependent pathway [29]. *SLC2A8* encodes glucose transporter type 8 (GLUT8), which mediates the transport of glucose and fructose. *GLUT8* overexpression or silencing significantly induces and blocks fructose uptake in cultured hepatocytes [30]. In addition to the above, genes that encoded major proteins in the electron transport chain and oxidative phosphorylation (*NDUFS4*, *TMEM126B*, *COQ4*, *LHPP*, *OXA1L*, *DMAC2L*, *COQ8A*) were also shared up-regulated in the two types of pandas (Table S15). Oxidative phosphorylation is a pervasive way for cells to use enzymes to oxidize glucose and produce adenosine triphosphate (ATP) [31]. Bamboo is rich in carbohydrates that occur as monosaccharides, disaccharides, and polysaccharides [32]. For grazing and browsing animals, carbohydrates have been considered a key energy source [32]. Previous studies have also shown giant pandas rely on starch and hemicelluloses in the plant for energy [33]. High expression of carbohydrate metabolism and energy production related genes in both pandas may improve the utilization of sugar components to fulfill their nutritional requirements from bamboo.

### Genes associated with lysine degradation

Liver is the principal site of lysine catabolism [34]. We found six liver genes (*AASS*, *ASH1L*, *NSD3*, *NSD1*, *KMT2C*, *KMT2D*, *SETDB1*) involved in lysine degradation showed adaptive convergence down-regulation (Table S16 and Fig. 5). *AASS* encodes α-aminoadipic semialdehyde synthase (AASS), which possesses bifunctional enzyme activity. AASS catalyzes the first two steps of the catabolism of L-lysine [35]. *ASH1L*, *NSD3*, *NSD1*, *KMT2C*, *KMT2D*, and *SETDB1* belong to histone lysine N-methyltransferase, which mediates the methylation of the amino acid lysine in the lysine degradation pathway and plays important roles in carnitine biosynthesis in mammals [36]. As an indispensable amino acid, lysine does not participate in transamination reactions and cannot be synthesized by mammalians [37]. Due to the importance of lysine in proteinogenesis and fatty acid metabolism, a lack of lysine can lead to many diseases including anemia [38], systemic protein-energy deficiency [39], and impaired fatty acid metabolism [40]. Lysine content in bamboo is much lower than in meat, or green plants [16]. Low expression of lysine catabolism-related genes would decrease the lysine requirement of the cell via a reduction of lysine catabolism through the saccharopine pathway [41], which may help both panda species survive on such a low-lysine bamboo diet.

### Genes associated with lipid metabolism

Genes associated with lipid and cholesterol metabolism were found down-regulated in liver (*ABCG5*, *ABCA1*, *MED13*, *THADA*, *GPAM*) and pancreas (*LOC100477302* (*CYP7B1*)) samples of both panda species (Table S17). Expression level of *ABCG5* is an important determinant of intestinal cholesterol absorption efficiency [42]. It is also essential for the maintenance of cholesterol homeostasis. Humans and mice lacking *ABCG5* and *ABCG8* has a marked reduction in biliary cholesterol secretion [43]. *ABCA1* encodes ATP Binding Cassette Subfamily A Member 1, which involves in regulating cellular cholesterol homeostasis and high density lipoprotein (HDL) formation [44], and it has been reported that mutations in *ABCA1* would cause the accumulation of cholesterol esters in macrophages and an increased risk of atherosclerosis [45]. *MED13* is initially discovered plays a key role in the control of systemic energy homeostasis from the heart. Recent study showed that cardiac overexpression of MED13 in mice is associated with increased lipid uptake in liver [46]. Although mammals lacking THADA function have not yet been described, researchers found *THADA* knockout flies were obese and produced less heat than controls, reflecting the primary effect of altered THADA activity and calcium signaling on lipid metabolism [47]. *GPAM* also known as *GPAT1*, which acts on the first step in the glycerophosphate pathway [48]. The glycerophosphate pathway is the main pathway for most TG synthesis [48]. Oxysterol 7α-hydroxylase (CYP7B1) is also a mitochondrial P450 enzyme. It participates in bile acid biosynthetic pathway of extrahepatic tissues, which converts cholesterol to bile acids [49]. Besides, 27-hydroxycholesterol (27HC) is an abundant oxysterol metabolized by CYP7B1. Previous research has found that elevations in 27HC via the deletion of CYP7B1 can cause exaggerated atherosclerosis [50].

Bamboo is a high-fiber and low-nutrition/lipid food [51]. Previous studies have shown a cholesterol-rich diet induces the up-regulation of genes related to cholesterol and fatty acid homeostasis [52, 53]. The down-regulation of lipid metabolism related genes in the two panda species may reflect the response to a long-term low-fat diet. Alternatively, adaptive convergence expression of these genes may result in low lipid metabolism in both panda species. It would indicate that both pandas may not be able to effectively absorb and utilize lipid from high-lipid meat.

### Regulatory mechanisms of gene expression

Epigenetic modifications play significant roles in phenotypic plasticity adaptive evolution [52, 54]. Research showed that different epigenetic regulatory patterns in p53 between the abutting chalk and basalt mole rat populations was associated with adaptive ecological sympatric speciation [55]. In our study, the convergent expression of nutrient metabolism genes may be regulated by DNA methylation, which including mostly lysine degradation and lipid metabolism related genes in the red panda and carbohydrate metabolism related genes in the giant panda. Convergent expression of nutrient metabolism-related genes is important for both pandas to adapt to a specialized bamboo diet. Recent studies indicated that epigenetic variation in natural populations can be independent from genetic variation, and that in some cases environmentally induced epigenetic changes may be inherited by future generations [19]. According to this principle, we propose that the epigenetic modifications of genes related to nutrition metabolism can also be inherited stably with the times, representing the long-term adaptability of both pandas to bamboo diet. Our research expands understand of DNA methylation influence on biological evolution and environmental adaptation.

Besides DNA methylation, transcription factor also plays an important role in regulating gene expression [56]. Different from the red panda, the core gene of the lysine degradation pathway (AASS) doesn’t exhibit hyper- or hypo-methylation modification in the giant panda. Dual-luciferase reporter assay showed that NR3C1 functions as a transcriptional activator in AASS transcription through the binding to AASS promoter region. Transcription factor is involved in organism evolution and the development of phenotypic variations [57]. The different regulation mechanisms of the AASS expression in both pandas indicated complex mechanisms underlying phenotypic convergence and adaptation to a specialized bamboo diet.

## Conclusions

In summary, our comparative transcriptome analyses of the liver and pancreas in red and giant panda species and non-herbivorous mammals demonstrated that a strict bamboo diet resulted in similar expression shifts in the two obligate bamboo-feeders. While more detailed functional studies of all the selected candidate genes will be necessary to confirm the roles of individual genes, the comparative analysis of both pandas provides insights into obligate herbivory-related genetic adaptations, such as high carbohydrate metabolism, low lipid metabolism and lysine degradation. Convergent expression of those nutrient metabolism-related genes in both pandas was an intricate process and subjected to multi-level regulation, including DNA methylation and transcription factor. Our research expands understand of DNA methylation and transcription factor influence on biological evolution and environmental adaptation.

## Methods

### Animal tissue collection

All giant and red panda samples were provided by Chengdu Research Base of Giant Panda Breeding in Chengdu and China Research and Conservation Center for the Giant Panda at Wolong, Sichuan Province, China. All giant and red panda samples were sourced from individuals that did not die for the purpose of this study (e.g. disease or accident) and the cause of death was unrelated to organs sampled. In red panda, liver and pancreas samples were obtained from six captive red panda adults (sample ID: afu_1025, afu_0708, afu_0429, afu_0527, afu_0601, afu_1219). Individual afu_1025 died of lung cancer. The cause of death of afu_0708, afu_0429, afu_0527, afu_0601 and afu_1219 was unknown. According to the autopsy report, individuals afu_0527, afu_0601 and afu_1219 had lung lesions. In giant panda, liver and pancreas samples were obtained from six wild giant panda adults (sample ID: aml_PP, aml_YS, aml_CC, aml_HT, aml_DN, aml_SE). These six adult giant pandas were seriously injured when found during other ecological investigations, and unfortunately rescue attempts were ineffective and died from their injuries. None of liver and pancreas tissues sampled exhibited pathological changes.

Four adult ferret individuals (sample ID: mpf_S1, mpf_S2, mpf_S3, mpf_S5) were bought from Wuxi Kuboyi Pet Products Co., LTD (China). All ferrets were killed by pentobarbital overdose and then liver and pancreas samples were taken for RNA-seq. The study is reported in accordance with ARRIVE guidelines.

### RNA extraction

Liver and pancreas tissue samples were immediately stored at -80℃ once obtained. Each organ (covering different structures/cells) was dissected and homogenized prior to RNA extraction. Total RNA was extracted using TRIzol reagent (Invitrogen, Carlsbad, CA, USA) according to the manufacturer’s instructions. All animal procedures performed in this research were in accordance with the ethical standards of Ethics Committee of College of Life Sciences, Sichuan University (Ethical Approval Number: SCU220802001).

In order to determine gene expression shifts in response to an exclusive bamboo diet, the liver and pancreas RNA-seq libraries of five Eutherian mammal species (human, dog, mouse, rat, cat) were downloaded from the NCBI Short Read Archive (SRA) database (Table 1). All samples were sourced from adult individuals. According to the NCBI SRA database, the individuals that supplied samples were generally healthy when tissues were collected. We selected RNA-seq libraries using the following criteria: (1) Genome sequences and gene set were available from NCBI databases; (2) Species closely related to the two panda species, but with different diet and similar digestive tract; (3) Species represented by at least duplicated biological replicates; and (4) Clean data was high-quality.

### RNA library preparation and sequencing

Sequenced libraries of giant panda, red panda and ferret samples were prepared using NEBNext® UltraTM RNA Library Prep Kit for Illumina® (NEB, USA) following the manufacturer’s instructions. Index codes were added to attribute sequences to each sample. After column purification, the quality of the resulting libraries was assessed on the Agilent Bioanalyzer 2100 system. The total RNA in aml_DN liver, aml_PP pancreas, aml_SE liver, aml_SE pancreas, afu_1219 liver, afu_1025 pancreas, afu_0429 liver, afu_0429 pancreas, mpf_S1 pancreas and mpf_S5 liver was degraded and did not meet the standard of library construction. The library preparations of other giant panda, red panda and ferret samples were sequenced on the Illumina HiSeq2000 platform. The reads are available from the NCBI Sequence Read Archive with BioProject accession number PRJNA612421. Description of the total 49 RNA-seq libraries for eight mammals used in this study is provided in Table S1.

### RNA-seq read mapping

Except for the red panda and giant panda, reference annotations of the other six mammal sequenced genomes were obtained from Ensemble, release 98 (Table S1). We used the red panda reference genome and reference annotation downloaded from DNA ZOO (https://www.dnazoo.org/). The red panda genome from DNA ZOO was reassembled using 3D-DNA pipeline [58] and reviewed using Juicebox Assembly Tools [59] based on the draft assembly ASM200746v1 (GCA_002007465.1) [16]. Low-quality reads and any adapter sequences were removed using NGS QC Toolkit [60] with a quality score of 20. High-quality reads that passed filter thresholds were mapped using HISAT2 [61]. Final efficiency of RNA-seq read alignments varied from 85.01 to 99.44% with species (Table S1). SAMtools was then used to convert the alignments in SAM format to BAM format. After reading in the reference annotations to count fragments, a count of all exons grouped by gene was calculated by featureCounts [62].

### Definition of orthologous genes

We performed an extensive orthologous gene comparison to investigate the expression level differences between obligate bamboo-eating pandas and other mammal species. Only the longest protein sequence was retained for each unique gene. Orthofinder 2.3.7 determined [63] one-to-one (1:1) orthologues between species within the liver and pancreas by using the reciprocal best hit method in BLASTp, with an E value cutoff of 1e-5. Orthologous gene ID and symbols of giant panda were used as proxies for following description of genes.

### Phylogenetic analysis

We identified 9,219 single-copy genes from the eight species. The amino acid sequences of all orthologous genes were aligned and concatenated to construct the phylogenetic tree. The maximum likelihood (ML) tree was performed in RAxML [64], with 100 bootstrap replicates under the PROTGAMMAJTT model. This process followed the python3 script in GitHub (https://github.com/dongwei1220/EasySpeciesTree).

### Expression level normalization

For cross-species comparison, the RNA-seq experiments will result in not only different gene lengths but also different sequencing depths. Gene expression levels for 1:1 orthologues were normalized using GeTMM (Gene length corrected TMM) [65]. This method combined gene length correction with the normalization procedure trimmed mean of M-values (TMM) (applied in edgeR) to obtain expression levels of orthologous genes comparable between species. We constructed gene expression matrices of liver and pancreas samples separately with each column presenting a sample and each row presenting the expression of an ortholog. Low expressed genes were filtered to include only genes expressed greater than 0 counts in the samples of the same species. We then defined each species as a group and a set of scaling factors were computed using TMM to normalize the library sizes. Normalized GeTMM values were used in downstream analyses. We assessed the data quality by comparing CV (ratio of the standard deviation to mean) of gene expression data before and after normalization. The CV of normalized data was lower than nonnormalized data (Figure S4), which indicated the bias due to species and biological replicates, which was reduced after normalization.

### Principal component analysis (PCA)

Normalized gene expression matrices of each tissue were log2 transformed. The PCA was performed on these transformed data using the ‘prcomp’ function in the R package ‘stats’.

### Correlation analysis between species

The liver and pancreas expression matrices of n rows (gene) by m columns (samples) were constructed. We calculated the Spearman correlation of each sample using the function “cor” in R, and the function ‘heatmap.2’ in package ‘gplots’ was used to plot the results.

### Differentially expressed genes

The GeTMM values were used to analyze gene expression differences with generalized linear models (GLM) in edgeR. Giant panda and red panda samples were compared to other non-panda samples separately. Significant DEGs equaled |log_2_FC|< 1 and Benjamini and Hochberg FDR-adjusted *P*-value < 0.05. DEGs that were shared between each panda species and the other non-herbivorous species were identified as convergent expression genes. DEGs were categorized and clustered by columns using the function ‘pheatmap’ in package ‘pheatmap’. We then performed gene ontology and pathway enrichment analysis of DEGs by using ‘enricher’ function in the ‘clusterProfiler’ package in R [66], with all genes of giant panda as the reference gene set.

### Real-time quantitative PCR

Experimental C57BL/6 J mice (8 weeks, *n* = 4) and Wistar Han rats (8 weeks, *n* = 4) were purchased from Chengdu Dossy Experimental Animals Co., Ltd. We collected the pancreas samples from mice and rats followed the ‘Guide for the care and use of laboratory animals’. Total RNA was extracted using M5 Universal RNA Mini Kit (Mei5 Biotechnology, China) according to the manufacturer’s instructions. The RNA of giant pandas, red pandas, ferrets, mice and rats were used for real-time PCR assay (Table S18). The isolated RNA was converted to double-stranded cDNA using M5 Sprint qPCR RT Kit (Mei5 Biotechnology, China). After the cDNA synthesis, quantification of 10 mRNA levels was conducted by real-time PCR performed on a CFX96 real-Time PCR Detection System. The expressions of 5 shared adaptive convergence DEGs associated with carbohydrate metabolism and respiratory electron transport related-genes were verified. All the primer sequences used for amplification of those 5 mRNAs were shown in Table S19.

The total volume of 10 μl reaction mix for the real-time PCR contained 5 μl 2X M5 HiPer SYBR Premix EsTaq (with Tli RNaseH) (Mei5 Biotechnology, China), 0.2 μl forward primer (10 pmol/μl), 0.2 μl reverse primer (10 pmol/μl), and 1 μl cDNA severed as a template and 3.6 μl ddH2O. Negative controls containing water as template were also included in each run. The cycling conditions were as follows: 1 cycle of 95 °C for 30 s; 40 cycles of 95 °C for 5 s, 60 °C for 30 s. Then, the expression levels of the mRNAs above were analyzed using the relative quantification (delta-Ct method). The housekeeping gene, GAPDH, was included as internal controls in all RT-qPCR runs. Expression of each gene verified by RT-qPCR were showed in Table S20.

### DNA methylation of convergently expressed nutrition metabolism-related genes in the promoter region

In order to explore the molecular mechanisms underlying epigenetic regulation of convergently expressed nutrition metabolism-related genes in both panda species, whole-genome methylation sequencing on liver and pancreas tissues of adult giant pandas and red pandas were performed, and the corresponding tissue methylation data of humans and mice were downloaded from SRA database for comparative analysis (Table 2). The DNA purity and concentration of afu_1025 liver, afu_0708 liver, afu_1025 pancreas, afu_0527 pancreas, afu_1219 pancreas, aml_PP pancreas and aml_SE liver was substandard. They were removed from further analysis. The processes for library preparation, bisulfite sequencing, and reads mapping were described in previous study [67]. For each sample, 109-251G clean base was generated after data quality control, and the final efficiency of BS-seq read alignments was ranged from 61.1 to 79.3%.

**Table 2:** Summary of collected samples and reads quality for WGBS

Scientific name	Sample ID	Organ	Clean base (G)	Mapping rate(%)	SRA accessions
*Ailurus fulgens*	afu_0601	Liver	119G	70.4%	SRR18502788
*Ailurus fulgens*	afu_1219	Liver	136G	76.6%	SRR18502787
*Ailurus fulgens*	afu_0429	Liver	126G	73.4%	SRR18502783
*Ailurus fulgens*	afu_0527	Liver	123G	77.7%	SRR18502782
*Ailurus fulgens*	afu_0601	Pancreas	220G	74.6%	SRR18502781
*Ailurus fulgens*	afu_0527	Pancreas	123G	79.3%	SRR18502780
*Ailurus fulgens*	afu_0708	Pancreas	129G	75.2%	SRR18502779
*Ailuropoda melanoleuca*	aml_CC	Liver	138G	75.7%	SRR13334621
*Ailuropoda melanoleuca*	aml_DN	Liver	129G	77.4%	SRR13286861
*Ailuropoda melanoleuca*	aml_HT	Liver	125G	65.2%	SRR13334613
*Ailuropoda melanoleuca*	aml_PP	Liver	118G	76.7%	SRR13334611
*Ailuropoda melanoleuca*	aml_YS	Liver	131G	70.9%	SRR13334610
*Ailuropoda melanoleuca*	aml_CC	Pancreas	138G	73.7%	SRR13334620
*Ailuropoda melanoleuca*	aml_DN	Pancreas	119G	71.4%	SRR13334617
*Ailuropoda melanoleuca*	aml_HT	Pancreas	130G	73.1%	SRR13334612
*Ailuropoda melanoleuca*	aml_SE	Pancreas	111G	75.0%	SRR13286860
*Ailuropoda melanoleuca*	aml_YS	Pancreas	135G	71.1%	SRR13334609
*Homo sapiens*	has_01	Liver	135G	63.2%	SRR10165496-SRR10165503
*Homo sapiens*	has_02	Liver	145G	61.1%	SRR10165528-SRR10165535
*Homo sapiens*	has_03	Liver	114G	61.9%	SRR10165698-SRR10165705
*Homo sapiens*	has_04	Pancreas	251G	76.4%	SRR8659897
*Homo sapiens*	has_05	Pancreas	211G	74.5%	SRR8659930
*Mus musculus*	mmu_01	Liver	109G	75.4%	SRR1534676
*Mus musculus*	mmu_02	Liver	135G	76.3%	SRR1534678

A negative correlation was generally occurred between promoter methylation and gene expression levels. We focused on the DNA methylation levels of all convergently expressed nutrition metabolism-related genes in the promoter region. The methylation level of the 1,000 bp promoter (the region from –1000 relative to the transcription start site) was calculated using the formula: Methylation level of promoter = ∑mC / ∑(mC + C). mC was the number of methylation reads in promoter and C was the number of unmethylation reads. Promoters had to contain at least 2 CpG sites which were covered by more than five reads. Except for the promoter region of the red panda *COQ8A* gene, all other genes met the above requirements. Differentially methylated promoters between giant panda and human, giant panda and mouse, red panda and human, and red panda and mouse were identified. The difference in methylation level between two groups was identified with a *P*-value < 0.05 using Two-tailed T-test.

### Gene clone, plasmids construction and luciferase assays

IN order to explore whether the down-regulated AASS that we had identified in giant panda liver was regulated by NR3C1, we performed luciferase assays. First, total RNA of giant panda live was extracted by using M5 Universal RNA Mini Kit (Mei5 Biotechnology, Co., Ltd) following the manufacturer's protocol. The extracted RNA samples were then used for the cDNA synthesis by using M5 Super plus qPCR RT kit (Mei5 Biotechnology, Co., Ltd). The giant panda NR3C1 was amplified by PCR using cDNA as template with the primers (Table 3), which were designed according to the sequences from giant panda genome. The PCR reaction system is 25 μl, which included 2.5 μl 10 × Taq Buffer (without Mgcl2), 2 μl dNTP Mixture (2.5 mM each), 0.5 μl of forward and reverse primer (10 μmol/L) respectively, 0.5 μl Taq DNA Polymerase (Vazyme) and 0.5 μl cDNA, added nuclear-free H_2_O to 25 μl.

**Table 3:** Primers for the PCR amplifications of *NR3C1* protein coding region

Primer	Primer sequence	Length	Anneal temperature	Extension time
GR-head-F	5’CTGATGATAATCTCTGATGGACTC3’	197 bp	55℃	12 s
GR-head-R	5’TTTGCTGCTTGGAATCTGACTG3’			
GR-F	5’ATGGACTCCAAGGAATCACTAAG3’	1048 bp	54℃	1 min
GR-1R	5’GTTGTTGAGAAAGGGATGCTGTATT3’			
GR-3F	5’CAGAGAAAGAGGCGAGTGAGAGTC3’	705 bp	58℃	42 s
GR-3R	5’TCCCACAAGTCAAGACCCCGTAAT3’			
GR-6F	5’CTCAGGGTGTCATTACGGGGTCT3’	963 bp	55℃	1 min
GR-8R	5’TCAATACTCATGGTCTTATCCAA3’			
GR-9F	5’TCTGTTCATGGTGTGAGTACCTC3’	690 bp	57℃	42 s
GR-9R	5’GTCATACCCTGCGTATAACACC3’			
GR-11F	5’AAAACCTTACTGCTTCTCTCTTC3’	578 bp	56℃	36 s
GR-11R	5’TATCACTCTCTACTCTTCCCACC3’			

Then, 30 mg liver sample of giant panda was used for DNA extraction according to the instructions of Tissue Genomic DNA Extraction Kit (Tiangen). 1,000 bp region upstream the AASS transcription start site was amplified based on the annotation information of giant panda genome (Primers: F 5’GTCTTGGGGCAATGGTCTA3’; R5’TAACAGGGTGTCCGTTCTG3’). The PCR reaction system is 50 μl, which included 25 μl 2 × Phanta Max Buffer, 1 μl dNTP Mix (10 mM each), 2 μl of forward and reverse primer (10 μmol/L) respectively, 1 μl Phanta Max super-fidelity DNA Polymerase (Vazyme) and 1 μl DNA, added nuclear-free H_2_O to 50 μl.

The sequence of AASS gene promoter region and NR3C1 protein coding region (Figure S5) were sent to company (Jin Kairui, Wuhan) for synthesis. The synthesized fragments contain restriction sites KpnI and XhoI. AASS promoter was cloned into pGL3-basic (Promega) and named AASS1000-pGL3-basic. NR3C1 was cloned into pCDNA3.1 (Invitrogen) and named pcDNA3.1-NR3C1. The orientations and the sequences of the inserts were verified by restriction digestion and sequencing.

293 cells were cultured at a density of 2 × 10^4^ cells/well in 96-well culture plates and co-transfected with 0.2 μg of the luciferase reporter construct and the internal control vector pRL-TK (Promega, Madison, WI) at a ratio of 20:1 (reporter construct: control vector) using LipofectamineTM 2000 (Invitrogen, Carlsbad, CA) according to instruction of the manufacturer. 5 h post-transfection, the transfection medium was removed and replenished with medium containing 6 μM of curcumin (Sigma-Aldrich, St. Louis, MO) solubilized in 100% dimethylsulfoxide (DMSO) (Sigma). 48 h post-transfection, luciferase activity was measured using the Dual-Luciferase® Reporter Assay System (Promega). Firefly luciferase activity was normalized to renilla luciferase activity in cells co-transfected with the reporter construct and the control vector.

## Supplementary Information

**Additional file 1:** **Table S1.** Description of the 49 RNA-seq libraries for eight mammals used in this study including giant and red pandas. **Table S2.** Normalized expression levels of shared 409 DEGs in liver samples of both panda species. **Table S3.** Normalized expression levels of shared 125 DEGs in pancreas samples of both panda species. **Table S4. **Significantly enriched GO categories for shared down-regulated DEGs in liver samples of both panda species. **Table S5.** Significantly enriched KEGG pathways for shared down-regulated DEGs in liver samples of both panda species. **Table S6.** Significantly enriched GO categories for shared up-regulated DEGs in liver samples of both panda species. **Table S7.** Significantly enriched KEGG pathways for shared up-regulated DEGs in liver samples of both panda species. **Table S8.** Significantly enriched GO categories for shared down-regulated DEGs in pancreas samples of both panda species. **Table S9.** Significantly enriched KEGG pathways for shared down-regulated DEGs in pancreas samples of both panda species. **Table S10.** Significantly enriched GO categories for shared up-regulated DEGs in pancreas samples of both panda species. **Table S11.** Significantly enriched KEGG pathways for shared up-regulated DEGs in pancreas samples of both panda species. **Table S12.** Transcription factors of shared down-regulated DEGs in both panda species. **Table S13.** Putative NR3C1 and promoter of AASS binding sites, identified by JASPAR. **Table S14.** RLU radio of firefly luciferase and renilla luciferase in five repeated experiments. **Table S15.** Shared up-regulated DEGs associated with carbohydrate metabolism and energy production in liver and pancreas of both panda species. The detailed information on gene function, involved pathways and annotations are from GeneCards databases (https://www.genecards.org/). **Table S16.** Shared down-regulated DEGs related to lysine degradation in liver of both panda species. The detailed information on gene function, involved pathways and annotations are from GeneCards databases (https://www.genecards.org/). **Table S17.** Shared down-regulated DEGs associated with lipid metabolism in liver and pancreas of both panda species. The detailed information on gene function, involved pathways and annotations are from GeneCards databases (https://www.genecards.org/). **Table S18.** The samples that used to do RT-qPCR. **Table S19.**  The primer of RT-qPCR. **Table S20.**  Relative expression levels of each gene verified by RT-qPCR.

**Additional file 2:** **Figure S1.** Volcano plot of differentially expressed genes in (A) liver and (B) pancreas. Each dot represents one gene. Red dots represent up-regulated differentially expressed genes, and blue dots represent down-regulated differentially expressed genes. Grey dots represent no significantly biased gene. The number at the top right represented the number of DEGs in pairwise comparison. **Figure S2. **The expression level of genes calculated by qRT-PCR.Y-axis represents relative expression levels of each gene by using 2^−ΔΔCT^ method. **Figure S3.** (A) The methylation degree in promoters for convergently expressed nutrition metabolism-related genes in liver samples. (B) The methylation degree in promoters for convergently expressed nutrition metabolism-related genes inpancreas samples. Y-axis represents methylation levels in promoters. * indicates *P* < 0.05 between the comparison, ** indicates *P* < 0.01 between the comparison, *** indicates *P* < 0.001 between the comparison, **** indicates *P* < 0.0001 between the comparison. **Figure S4. **Distributions of coefficient of variance of gene expression levels among liver and pancreas samples before and after normalization, for all 1:1 single-copy orthologues. Histogram was created with a density scale. A normal density curve was added to the histogram to make the distribution of CV more appealing. **Figure S5. **The ORF and amino acid sequence of giant panda NR3C1 gene.

## Data Availability

The datasets generated and analysed during the current study are available through NCBI under Bioproject ID PRJNA612421 (accession numbers SRR11301085- SRR11301096, SRR12158770- SRR12158773, SRR18502776- SRR18502788) (fastq format). These data will remain private until the related manuscript has been accepted. All other data generated in this manuscript are available from the corresponding author upon reasonable request.
