# Supplementary material for: Gene expressions between obligate bamboo-eating pandas and non-herbivorous mammals reveal converged specialized bamboo diet adaptation
Source: BMC Genomics. 2023 Jan 16;24:23. doi: 10.1186/s12864-023-09111-z (PMC9843897; doi:10.1186/s12864-023-09111-z)
Supplement: Supplementary file 2 — Additional file 2: Figure S1. Volcano plot of differentially expressed genes in (A) liver and (B) pancreas. Each dot represents one gene. Red dots represent up-regulated differentially expressed genes, and blue dots represent down-regulated differentially expressed genes. Grey dots represent no significantly biased gene. The number at the top right represented the number of DEGs in pairwise comparison. Figure S2. The expression level of genes calculated by qRT-PCR.Y-axis represents relative expression levels of each gene by using 2−ΔΔCT method. Figure S3. (A) The methylation degree in promoters for convergently expressed nutrition metabolism-related genes in liver samples. (B) The methylation degree in promoters for convergently expressed nutrition metabolism-related genes inpancreas samples. Y-axis represents methylation levels in promoters. * indicates P < 0.05 between the comparison, ** indicates P < 0.01 between the comparison, *** indicates P < 0.001 between the comparison, **** indicates P < 0.0001 between the comparison. Figure S4. Distributions of coefficient of variance of gene expression levels among liver and pancreas samples before and after normalization, for all 1:1 single-copy orthologues. Histogram was created with a density scale. A normal density curve was added to the histogram to make the distribution of CV more appealing. Figure S5. The ORF and amino acid sequence of giant panda NR3C1 gene. [file 12864_2023_9111_MOESM2_ESM.docx]

**
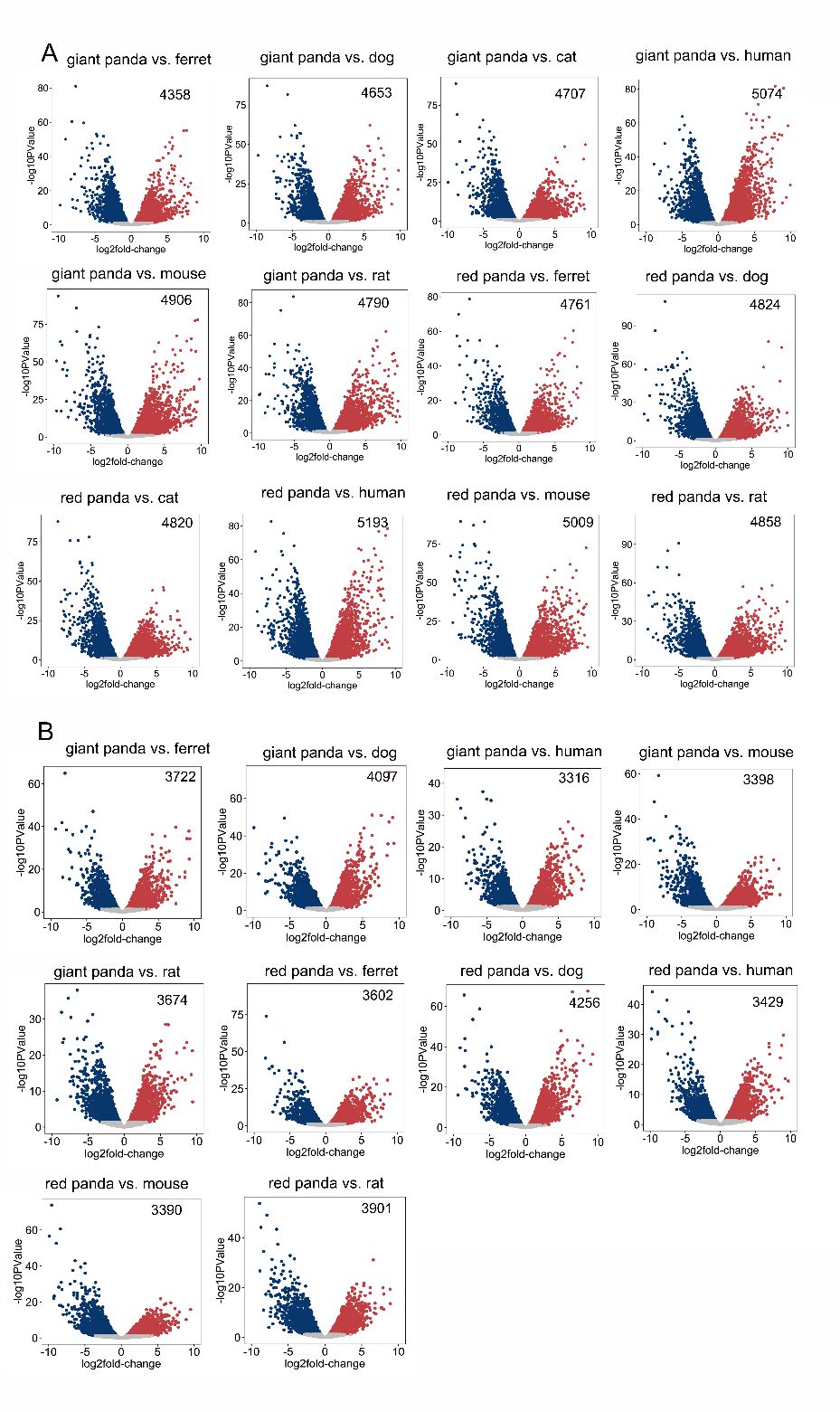
**

**Figure S1.** **Volcano plot of differentially expressed genes in (A) liver and (B) pancreas.** Each dot represents one gene. Red dots represent up-regulated differentially expressed genes, and blue dots represent down-regulated differentially expressed genes. Grey dots represent no significantly biased gene. The number at the top right represented the number of DEGs in pairwise comparison.


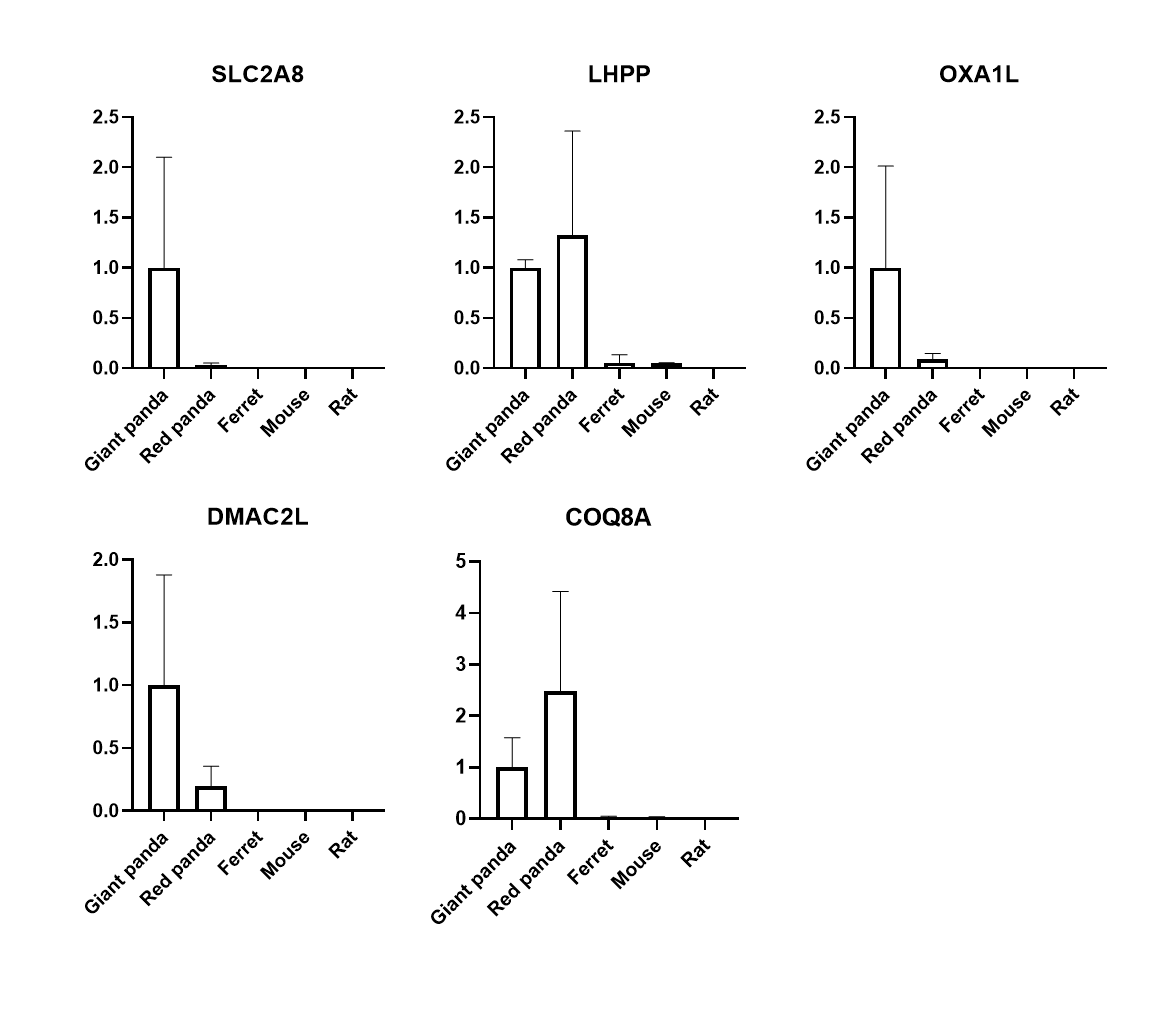


**Figure S2. The expression level of genes calculated by qRT-PCR.** Y-axis represents relative expression levels of each gene by using 2^−ΔΔCT^ method.


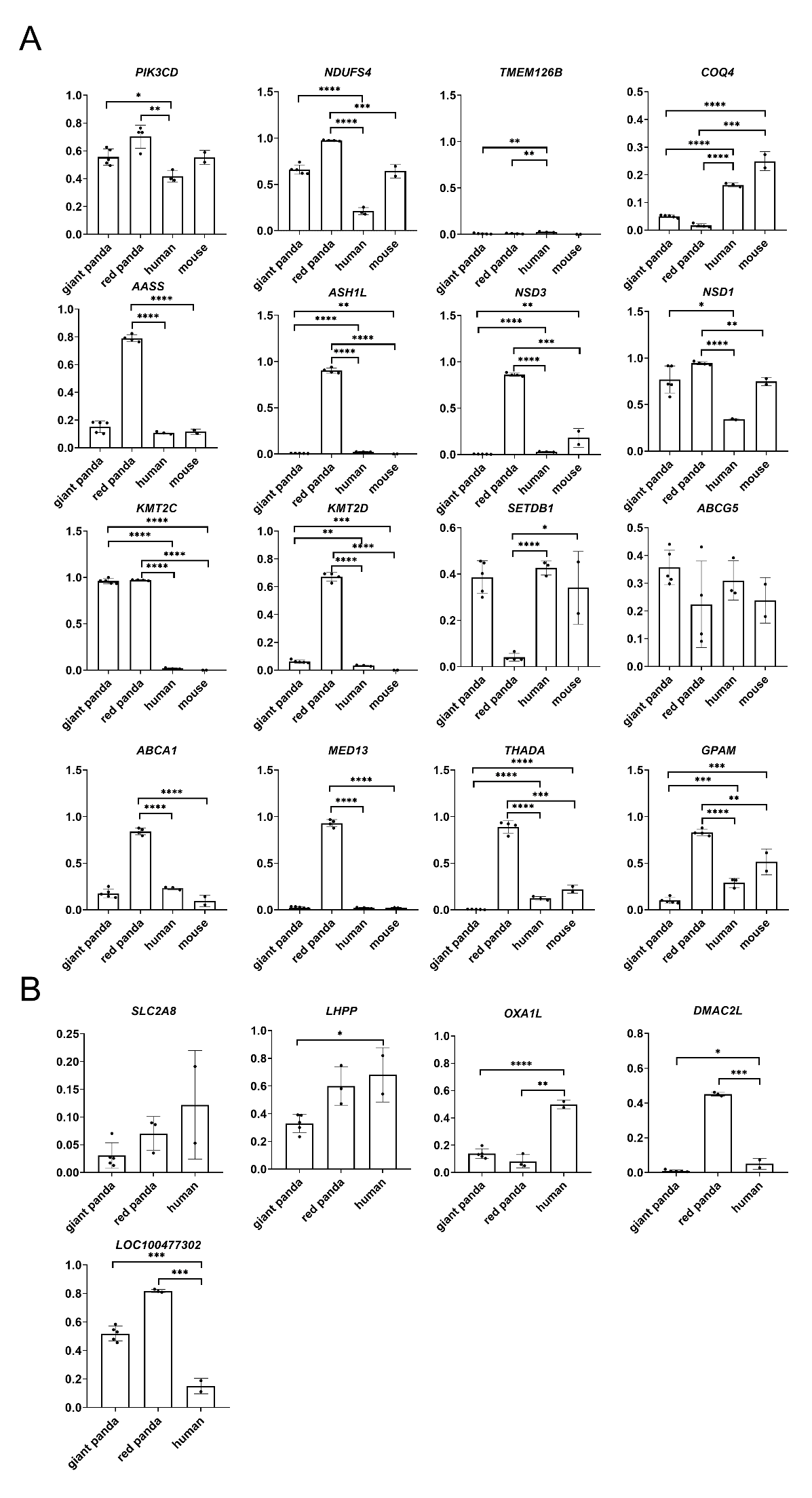


**Figure S3. (A) The methylation degree in promoters for convergently expressed nutrition metabolism-related genes in liver samples. (B) The methylation degree in promoters for convergently expressed nutrition metabolism-related genes in pancreas samples.** Y-axis represents methylation levels in promoters. * indicates P < 0.05 between the comparison, ** indicates P < 0.01 between the comparison, *** indicates P < 0.001 between the comparison, **** indicates P < 0.0001 between the comparison.

**
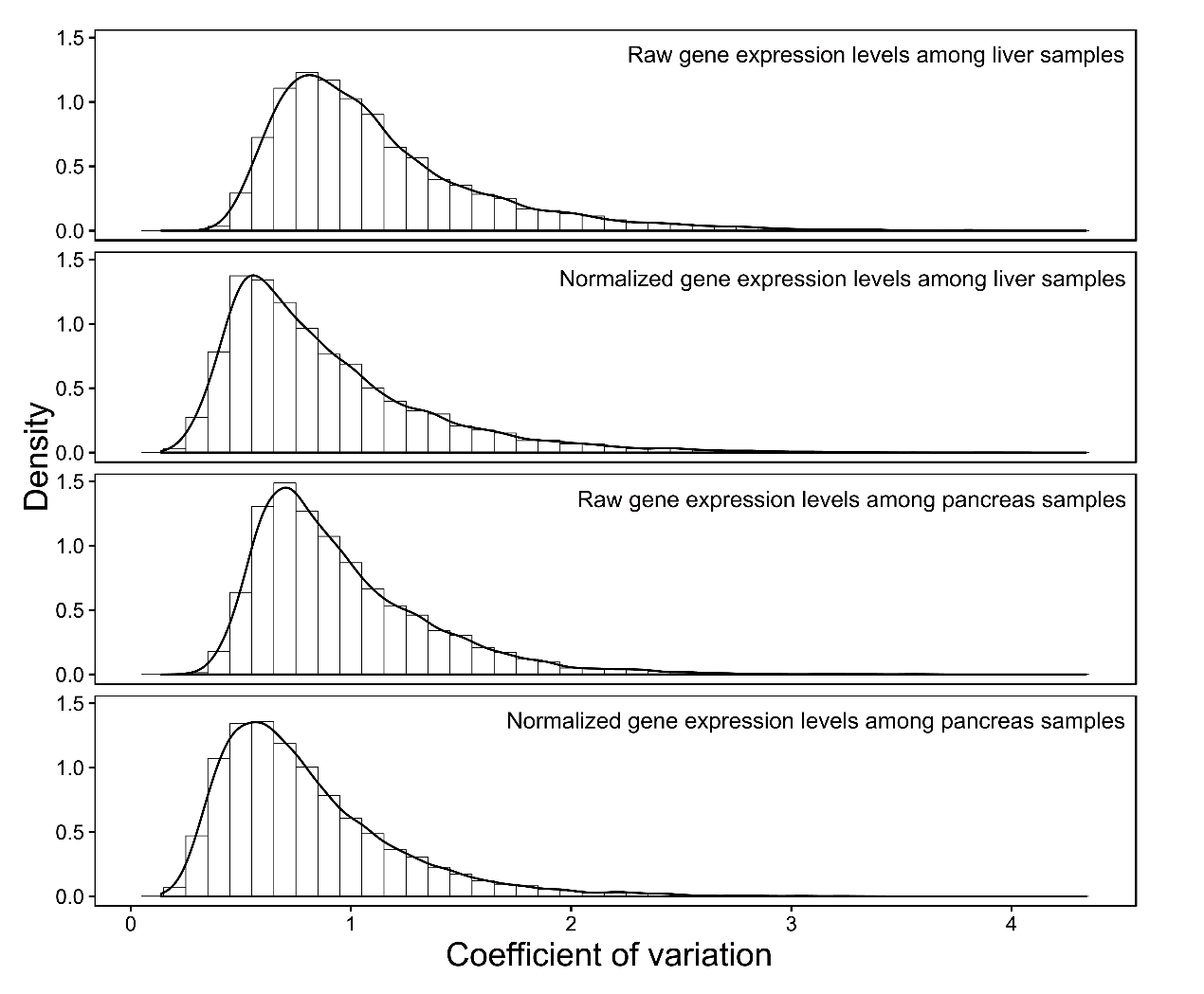
Figure S4. Distributions of coefficient of variance of gene expression levels among liver and pancreas samples before and after normalization, for all 1:1 single-copy orthologues.** Histogram was created with a density scale. A normal density curve was added to the histogram to make the distribution of CV more appealing.

**
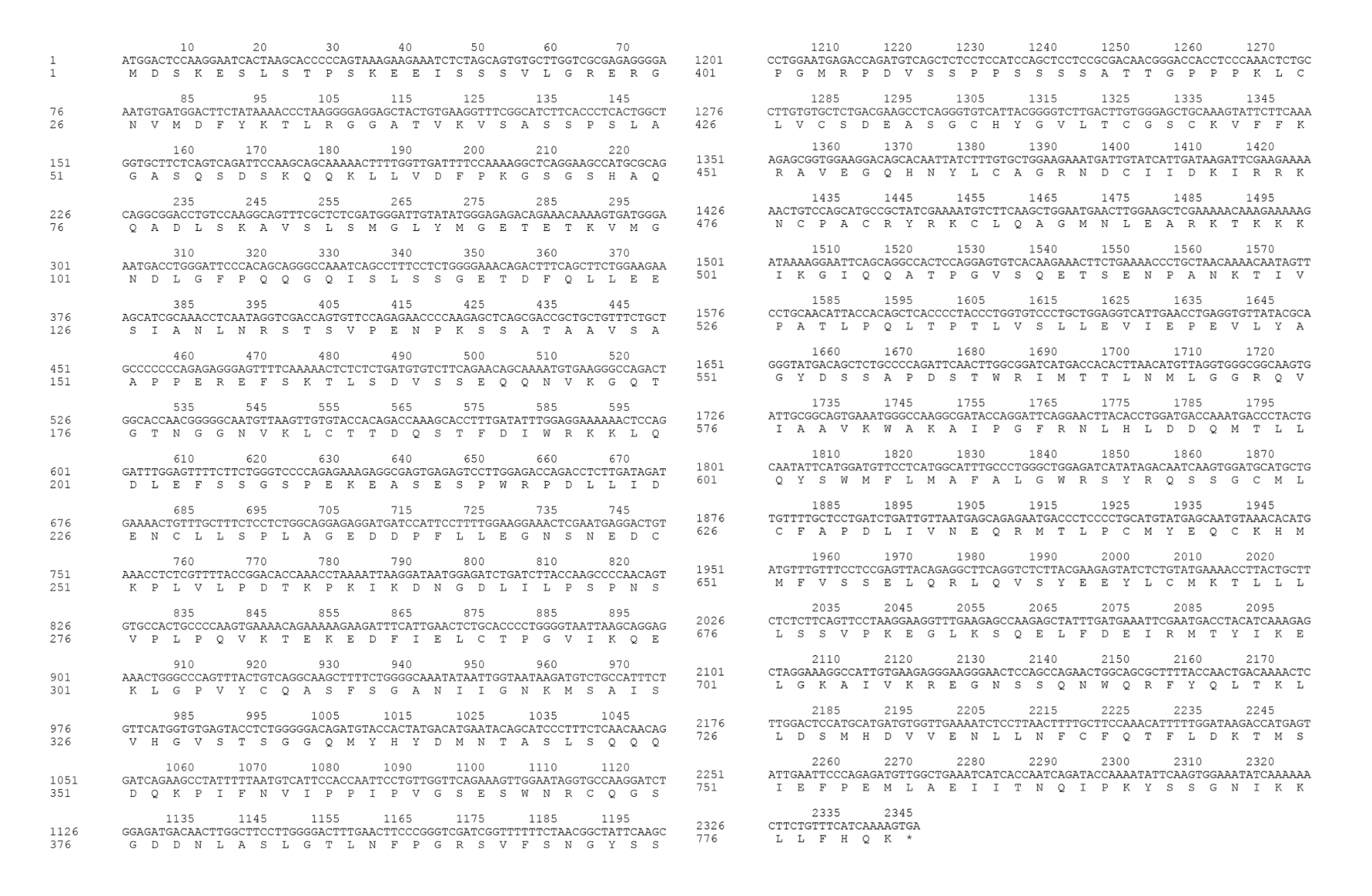
**

**Figure S5. The ORF and amino acid sequence of giant panda NR3C1 gene.**
